# Characterizing Focused-Ultrasound Mediated Drug Delivery to the Heterogeneous Primate Brain *In Vivo* with Acoustic Monitoring

**DOI:** 10.1038/srep37094

**Published:** 2016-11-17

**Authors:** Shih-Ying Wu, Carlos Sierra Sanchez, Gesthimani Samiotaki, Amanda Buch, Vincent P. Ferrera, Elisa E. Konofagou

**Affiliations:** 1 Department of Biomedical Engineering, Columbia University, New York, New York, USA; 2 Department of Neuroscience, Columbia University, New York, New York, USA; 3 Department of Radiology, Columbia University, New York, New York, USA

## Abstract

Focused ultrasound with microbubbles has been used to noninvasively and selectively deliver pharmacological agents across the blood-brain barrier (BBB) for treating brain diseases. Acoustic cavitation monitoring could serve as an on-line tool to assess and control the treatment. While it demonstrated a strong correlation in small animals, its translation to primates remains in question due to the anatomically different and highly heterogeneous brain structures with gray and white matteras well as dense vasculature. In addition, the drug delivery efficiency and the BBB opening volume have never been shown to be predictable through cavitation monitoring in primates. This study aimed at determining how cavitation activity is correlated with the amount and concentration of gadolinium delivered through the BBB and its associated delivery efficiency as well as the BBB opening volume in non-human primates. Another important finding entails the effect of heterogeneous brain anatomy and vasculature of a primate brain, i.e., presence of large cerebral vessels, gray and white matter that will also affect the cavitation activity associated with variation of BBB opening in different tissue types, which is not typically observed in small animals. Both these new findings are critical in the primate brain and provide essential information for clinical applications.

Blood-brain barrier (BBB) is a highly selective barrier in the cerebral endothelium that prevents neurotoxins from entering into the brain parenchyma while at the same time blocking the entry of diagnostic or therapeutic agents^1^,^2^. Focused ultrasound (FUS) in conjunction with microbubbles (1–10 μm) is currently the only way to open the BBB noninvasively, locally, and transiently for drug delivery and treatment for central-nervous-system (CNS) diseases^3^. It has been shown to treat glioblastoma^4^,^5^ and Alzheimer’s disease^6^,^7^ in small animal models. More applications are to be explored as various agents as small as magnetic resonance contrast agents (gadolinium, ~200 pm) to as large as viral vectors (~20 nm)^8^,^9^, nanoparticles (~100 nm)^10^,^11^, and stem cells (~10 μm)^12^ have be reported to pass the BBB with this technique. It has been translated to large animals such as non-human primates (NHP)^13^,^14^, with short-term (hours to 9 weeks) and long-term (4 to 20 months) safety confirmed with MRI, histology, and behavioral assessment^15^,^16^. This technique is under clinical trials to treat patients with malignant brain tumors^17^,^18^.

To ensure an effective and safe BBB opening for drug delivery and treatment, cavitation associated with BBB opening could be monitored^19^. This association of cavitation with BBB opening lies on the physical mechanism for BBB opening. Cavitation occurs in the vessels in the focal area during the FUS treatment, and the oscillating bubbles generated an acoustic signature reflecting both the strength and type of bubble activity^20^,^21^. If bubbles oscillate periodically without disruption, harmonic (bubble volumetric oscillation; n*f, where n = 1, 2, 3, … and f = excitation frequency) and/or ultraharmonic signals (bubble shell oscillation; n*f + f/2, where n = 1, 2, 3, … and f = excitation frequency) will be generated, a phenomenon known as stable cavitation. At higher pressures when bubbles oscillate violently and collapse rapidly, shock waves and microjets are generated with broadband emission known as inertial cavitation. Both stable and inertial cavitation applies mechanical stress onto the vessel walls^22^,^23^ which could disrupt the tight junctions and other processes of BBB and increase the permeability of capillary endothelium^24^.

Passive cavitation detection (PCD) served as a tool for real-time transcranial monitoring during FUS, and could serve as an online treatment evaluation complement to the post-operative MRI-based methods. Since its initial use during BBB opening in small animals^19^, PCD has been expanded to various types of contrast agents such as microbubbles of various sizes^25^, various shells^26^, and nanodroplets^27^ reporting a positive correlation between BBB opening and cavitation dose. A feedback control mechanism for BBB opening based on ultraharmonics and subharmonics was established in small animals^28^,^29^. The permeability and reversibility of BBB opening could also be predicted based on cavitation in mice^30^. Nevertheless, the translation of these findings with PCD monitoring to primates remains in question due to the thicker skull and heterogeneous brain parenchyma significantly attenuating the cavitation signals.

Since the preliminary study demonstrating the feasibility of transcranial cavitation detection in NHPs *in vivo*^14^ and evidence that both stable and inertial cavitation doses could be monitored in real time through the intact NHP and human skulls^31^, PCD monitoring has been steered towards clinical applications as it was the only technique assessing the treatment effectiveness in real time^32^. However, cavitation monitoring in NHP for assessing BBB opening was found to be highly variable^14^ and was not correlated with the BBB opening outcome such as opening volume^33^, unlike in small animals such as rodents that showed good correlation. We hypothesized that this discrepancy may be due to the heterogeneous and gyrencephalic nature of the primate brain (i.e., gray and white matter, and large cerebral vessels) in primates or the targeting parameters. Furthermore, information critical in future clinical applications such as the amount of drug delivered and its associated delivery efficiency has yet to be established with caviation *in vivo*. These issues can be of high importance and therefore essential in the pre-clinical evaluation of the technique in NHP.

Therefore, the aims of this study were to determine (1) how the cavitation activity correlated with the BBB opening volume, the amount of model drug (gadolinium) delivered through the barrier and its delivery efficiency; (2) how the heterogeneous primate brain (gray and white matter, large vessels) influenced the cavitation activity and the BBB opening outcomes; (3) the targeting accuracy and effect to the cavitation monitoring. Real-time transcranial cavitation monitoring of the stable and inertial cavitation doses was performed in four macaque monkeys during sonication through stereotactic targeting. For the quantitative analysis of BBB opening volume and drug delivery efficiency, T_1_-weighted (T_1w_) imaging and variable flip-angle (VFA) T_1_ mapping in magnetic resonance imaging (MRI) were performed 1 h following BBB opening. These opening outcomes were then correlated with the acquired acoustic monitoring findings in order to assess the PCD predictability value among animals. The effect of gray and white matter on BBB opening and cavitation was analyzed after tissue segmentation. The vasculature effect on cavitation monitoring was evaluated by targeting a region proximal to the medial cerebral artery (MCA) with a monotonically increasing pressure for PCD calibration. The effect of the incidence angle with respect to the skull on cavitation monitoring was evaluated by varying the targeting angle with PCD calibration as well.

## Results

### Variability in BBB opening and cavitation monitoring

**Figure 1:**
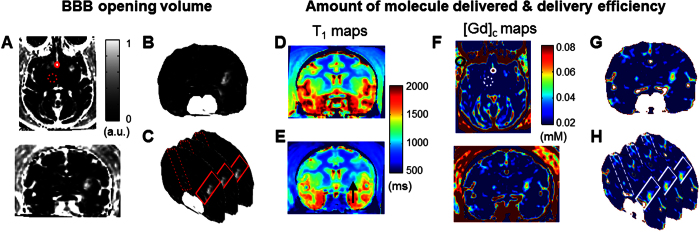
Pipeline for BBB opening volume quantification and drug delivery analysis based on MR image processing. (**A**) To quantify BBB opening volume, the contrast enhancement map (upper: horizontal slice, lower: coronal slice) were used after dividing the post-Gd T_1_ images by the pre-Gd images. Then, after applying the brain mask and the vessel mask in order to filter out the contrast enhancement outside of the BBB opening area (**B**), the opening volume was calculated by subtracting the VOI in the contralateral area (dashed rectangle) from the targeted area (solid rectangle) (**C**). On the other hand, the pre- Gd T_1,0_ (**D**) and post-Gd T_1_ maps (**E**) were used to quantify the amount of Gd delivered and its delivery efficiency since the Gd shorted the T1 time after diffused the BBB opening region (arrowhead). The Gd concentration map (**F**) (upper: horizontal slice, lower: coronal slice) was acquired by calculating the change of T_1_ time between pre- and post-Gd T_1_ maps. After applying the brain mask and excluding the Gd retention in the vessels by thresholding (**G**), the amount of Gd delivered was calculated by subtracting the VOI in the contralateral area (dashed rectangle) from the targeting area (solid rectangle) (**H**).

**Figure 2:**
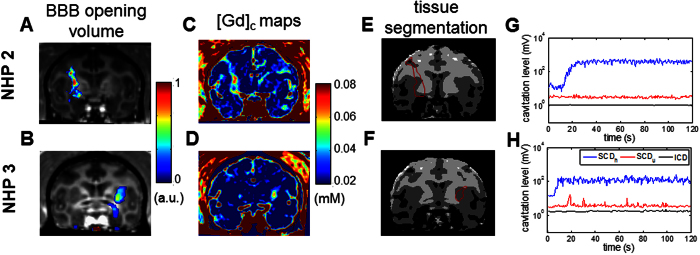
BBB opening with real-time acoustic cavitation monitoring in two NHPs. FUS-induced BBB opening in NHPs was visualized in T_1w_ images after overlaying the contrast enhancement onto the post-Gd T_1w_ image (**A,B**) (**A**: NHP 2 at 300 kPa; **B**: NHP 3 at 600 kPa). The corresponded Gd concentration map ([Gd]_c_) (**C,D**) showed the variation of Gd delivered in gray and white matter according to the tissue segmentation map based on the pre-Gd T_1_ map (**E,F**) (dark gray: gray matter, light gray: white matter, black: blood, white: CSF). The acoustic cavitation emission were recorded and calculated in real time, where SCD_h_ denotes stable cavitation dose with harmonics, SCD_u_ for stable cavitation dose with ultraharmonics, and ICD for inertial cavitation dose. (**G,H**). The BBB opening of NHP 2 and 3 showed an inter-animal variation and may be due to the skull.

With the FUS system setup targeting the basal ganglia (Supplementary Fig. S1), the BBB opening in NHP was achieved with the opening volume and delivery efficiency quantified following the pipeline in Fig. 1 through pre- and post-contrast (Gd, gadolinium) T_1w_ imaging and T_1_ mapping for Gd concentration ([*Gd*]_*c*_) as the Gd, a paramagnetic particle does not cross the BBB, perfused the BBB opening region and shortened the relaxation time of the tissue. Figure 2 showed representative BBB opening findings in two NHPs with acoustic monitoring of the cavitation dose (SCD_h_: stable cavitation dose with harmonics, SCD_u_: stable cavitation dose with ultraharmonics, ICD: inertial cavitation dose with broadband emission). BBB opening was revealed in both T_1w_ imaging (Fig. 2A,B) and the [*Gd*]_*c*_ map (Fig. 2C,D) in both gray and white matter (Fig. 2E,F) after a significant increase of SCD_h_ (Fig. 2G,H). In NHP 2 (300 kPa on the right putamen), an opening volume of 298 mm^3^ was achieved with the highest [*Gd*]_*c*_ in the opening area reached 0.07 mM, and the amount of Gd delivered was 10.7 nmol. The BBB opening volume was 393 mm^3^ in NHP 3 (600 kPa on the left putamen) with the highest [*Gd*]_*c*_ reached 0.06 mM, and the amount of Gd delivered was 8.7 nmol. The [*Gd*]_*c*_ was higher in gray matter than that in white matter in the BBB opening area. While comparing to the Gd retention of the unsonicated tissue (Table 1), the [*Gd*]_*c*_ at the center of the opening was higher than that in the unopened brain parenchyma (0.01–0.02 mM), similar to that of the muscle (0.06–0.07 mM), and lower than that of the vessel (0.14–0.15 mM).

**Figure 3:**
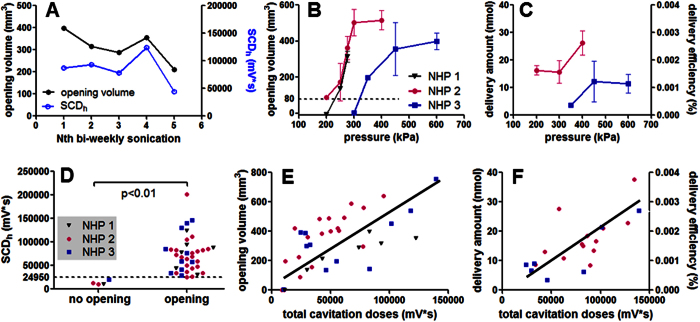
Cavitation monitoring in assessing the BBB opening and drug delivery. (**A**) In order to study the intra-animal variation, NHP 1 was sonicated with the same targeting (putamen) and acoustic parameters (275 kPa) in a bi-weekly basis five times. FUS-induced BBB opening was performed in three NHPs with the opening volume (**B**), and the drug delivery analysis was performed in two NHPs for the amount of Gd delivered and the delivery efficiency (**C**). NHP 1 to NHP 3 were sonicated 7, 12, and 24 times, respectively, and the error bar represents standard deviation. Both (**B)** and (**C**) showed an inter-animal variation as the BBB opening threshold for NHP 1 and 2 were lower than that for NHP 3. Quantified cavitation doses (SCD_h_: stable cavitation dose with harmonics, SCD_u_: stable cavitation dose with ultraharmonics, ICD: inertial cavitation dose) were correlated with the BBB opening outcomes. The SCD_h_ can be used to detect the effectiveness of BBB opening (**D**). For a quantitative assessment, the opening volume (**E**), the amount of delivered Gd and delivery efficiency (**F**) was positively correlated with the total cavitation dose (SCD_h_ + SCD_u_ + ICD). (The R^2^ of the linear fitting in (**E**) for NHP 1 to 3 and all experiments across animals was 0.81, 0.63, 0.50, and 0.47, respectively; that in (**F**) for NHP 2, 3, and all experiments across animals was 0.52, 0.71, and 0.61, respectively.)

Both intra- and inter-animal variation in BBB opening were observed in the quantitative results (Fig. 3). In repeated sonication in NHP 1 (275 kPa at putamen, Fig. 3A) to estimate intra-animal variation, the coefficient of variation in the opening volume was 22% (ratio of the standard deviation to the mean). Inter-animal variation was observed in the pressure threshold for BBB opening after applying an estimate of 50% pressure increase to compensate the skull attenuation. As shown in Fig. 3B, the pressure threshold for NHP 1 and 2 was lower (250 kPa) than that in NHP 3 (350 kPa) with the same range of vital signs during sonication (Supplementary Table S1). This discrepancy was also observed in the [*Gd*]_*c*_ map analysis performed in NHP 2 and NHP 3 (Fig. 3C), showing that the amount of Gd delivered and the delivery efficiency in NHP 2 was higher than that in NHP 3 using the same pressure. This inter-animal variation may be due to the difference in skull and tissue attenuation, and can be corrected through simulation predictions based on the same animal’s CT^34^. Nevertheless, an opening volume of 400 mm^3^ and 15 nmol of Gd delivered (0.0015% of delivery efficiency) could be achieved.

Cavitation monitoring characterized BBB opening without significant intra- and inter-animal variation, and could be used to predict BBB opening volume, amount of delivered Gd and its delivery efficiency. The cavitation monitoring was positively correlated with the BBB opening volume given the intra-animal variation (Fig. 3A), suggesting the possibility of using cavitation monitoring as a feed-back loop to control the FUS treatment in NHPs. Moreover, the SCD_h_ (sensitive to bubble activity at low pressures) was found to be an indicator of the effectiveness of the FUS procedure among animals (Fig. 3D, p < 0.01). The total cavitation doses (SCD_h_ + SCD_u_ + ICD) was positively and linearly correlated with the opening volume (Fig. 3E, R^2^ = 0.47), amount of delivered Gd and delivery efficiency (Fig. 3F, R^2^ = 0.61), and could serve as a surrogate for MRI-based treatment evaluation. No significant difference was found among animals, while the R^2^ of each individual varied. The total cavitation doses was adopted since it best correlated with the opening outcomes as the SCD_h_ and SCD_u_ may reach a plateau at high pressures while the ICD was detected^31^. Transcranial cavitation monitoring showed insignificant intra-animal variation may be because the recorded cavitation signal reflected the *in situ* pressure after attenuation.

### Effect of brain heterogeneity

**Figure 4:**
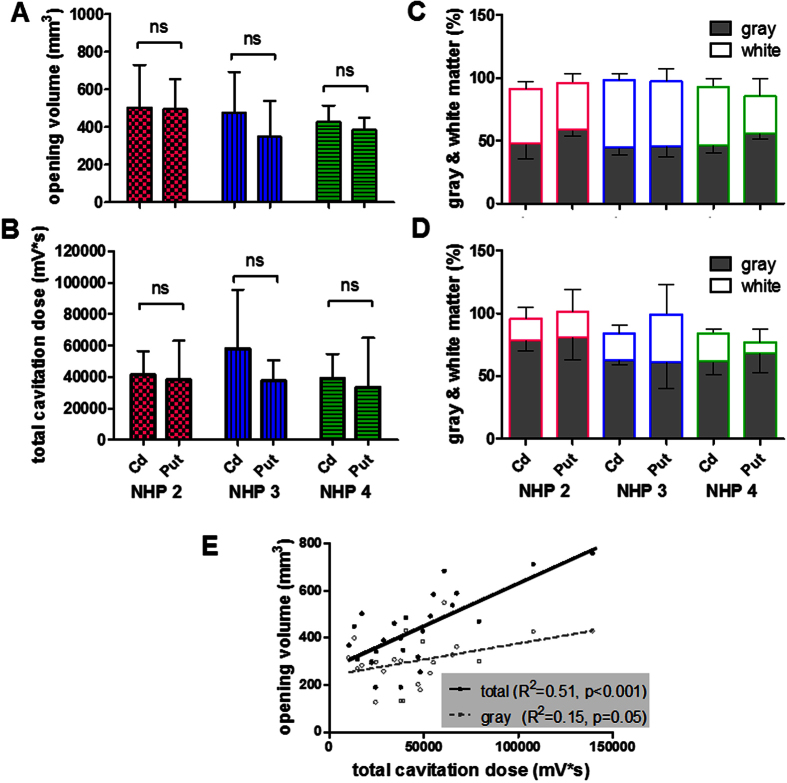
Effect of gray and white matter. FUS was applied in the caudate and putamen in three animals causing a BBB opening volume close to 400 mm^3^ (300 kPa for NHP 2, 450 kPa for NHP 3 and 4). The opening volume was shown in (**A**), and the total cavitation dose in (**B**). Based on the tissue segmentation, the gray-and-white matter composition in the sonicated area (**C**) and in the BBB opening area (**D**) were calculated. The correlation of total cavitation dose to the BBB opening volume was calculated in (**E**), in which it was better correlated with the total opening volume than with the volume in gray matter.

The effect of brain heterogeneity on BBB opening, drug delivery, and cavitation monitoring was separated into two parts: the effect of gray and white matter, and the effect of large vessels. First, in studying the effect of gray and white matter (Fig. 4), three animals were sonicated at the caudate nucleus and putamen at pressures causing a BBB opening volume of 400 mm^3^ (300 kPa for NHP 2, and 450 kPa for NHP 3 and 4 due to the inter-animal variation). The opening volume in the caudate nucleus was the same or slightly smaller than that in the putamen in the same animal without statistical significance (Fig. 4A), and the cavitation response revealed the same trend (Fig. 4B). Although the gray-and-white matter composition in the sonicated area was about 1:1 (Fig. 4C), the gray matter contributed 68.8% of the BBB opening in comparison to 21.4% for the white matter (Fig. 4D), meaning that the gray matter had the probability of BBB opening three times higher than that of the white matter. In order to investigate the capability of cavitation monitoring on the gray and white matter, their opening volume were delineated for correlating with the total cavitation dose. As shown in Fig. 4E, a better correlation of cavitation dose to the total opening volume was found (R^2^ = 0.51, p < 0.001) compared with the volume of gray matter only (R^2^ = 0.15, p = 0.05), suggesting that the cavitation was detected in both the gray and white matter and caused BBB opening in both.

**Figure 5:**
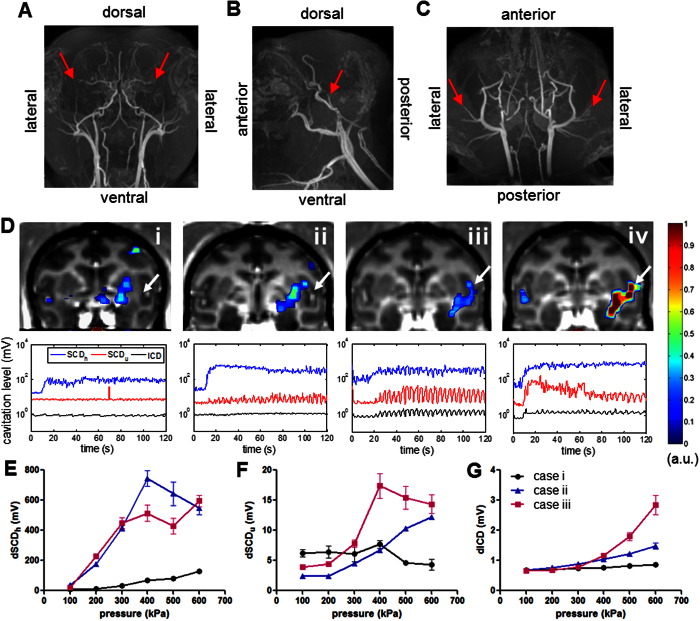
Vasculature effect. MR angiography (MRA) in NHP in (**A**) coronal, (**B**) sagittal, and (**C**) horizontal view, and the middle cerebral artery (MCA) was indicated by an arrowhead. (**D**) Four BBB opening cases targeting regions proximal to the MCA (arrowhead) at 450 kPa in NHP 4, where the upper row showed the opening volume overlaying onto the post-Gd T_1w_ images and the lower row was the cavitation response along the sonication time. PCD calibration (**E–G**) after BBB opening in cases i-iii was performed in order to assess the cavitation level at different pressures (10 pulses per pressure) with targeted regions near or include the MCA (E: SCD_h_, F: SCD_u_, G: ICD), and the errorbar represented the standard deviation of the 10 sonications. Note that the opening volume for case i to iv was 309, 469, 443, and 758 mm^3^ and angle of incidence to the skull: 24°, 18°, 35°, 41°, respectively. The cavitation level varied as the targeted region approached the MCA that was correlated with the opening volume but was found to be independent of the incidence angle.

Second, the effect of large vessels (diameter ≥1 mm) in the BBB opening and cavitation monitoring were investigated by targeting a region (putamen) close or including the middle cerebral artery (MCA) in NHP 4. As shown in the MRA (Fig. 5A–C), the NHP brain is rich in cerebral blood vessels of different sizes. When applying FUS at 450 kPa, the BBB opening was found to be successful regardless of the distance to MCA in the four cases in Fig. 5D (opening volume: 309, 469, 443, and 758 mm^3^ and angle of incidence to the skull: 24°, 18°, 35°, 41° for case i to iv, respectively). However, the cavitation response was significantly enhanced as the focus drew closer to the MCA. Interestingly, the spontaneous SCD_u_ and ICD were found to be reliably detected when targeting a region including MCA, and a periodicity of the cavitation level was similar to the breathing rate in certain cases where large vessels were in the focus. This increased cavitation response was also observed in the PCD calibration assessing the cavitation level (the cavitation dose of one single pulse) at different pressures (Fig. 5E–G) after the BBB opening in Fig. 5D. The ICD increased monotonically with pressure (Fig. 5G) while the SCD_h_ (Fig. 5E) and SCD_u_ (Fig. 5F) increased until a plateau was reached at higher pressures. Furthermore, although the opening volume in cases ii and iii were similar, the cavitation level of SCD_h_, SCD_u_, and ICD in the PCD calibration followed a different variation. The SCD_h_ was slightly lower at pressures above 300 kPa while the SCD_u_ and ICD were higher in case iii (MCA in the focus) compared to case ii (Fig. 5D).

### Targeting accuracy and the effect to cavitation monitoring

**Figure 6:**
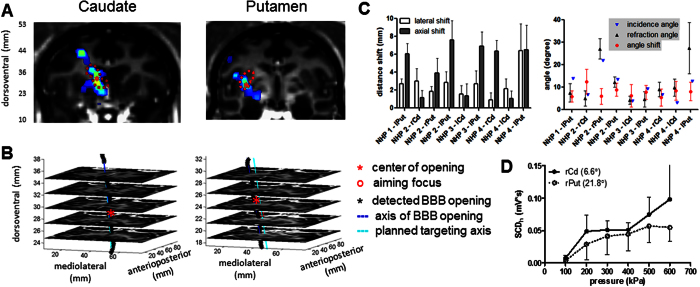
Targeting accuracy and the effect on cavitation monitoring. (**A**) Visualization of targeting in the caudate nucleus (dashed contour in the left column) and the putamen (dashed contour in the right column) and the BBB opening by overlaying the contrast enhancement onto the post-Gd T_1w_ image, where * denotes the centroid of BBB opening. (**B**) Targeting trajectory and the opening trajectory showed in the stacked horizontal slices. (**C**) Target shift in distance (top) and angle (down), where lPut represents left putamen, rPut for right putamen, lCd for left caudate, and rCd for right caudate. (**D**) The effect of incidence angle to the cavitation response in NHP 2, where the legend denotes targeted structure and the incidence angle. The error bar represents standard deviation.

Following targeting planning in the caudate and putamen, the resulting BBB opening was visualized in brain in the 2D coronal plane (Fig. 6A), in the stacked 2D horizontal planes (Fig. 6B) and in 3D through the skull (Supplementary Fig. S2). The targeting accuracy was quantified in four animals (Fig. 6C) based on the defined FUS orientation (axial and lateral distance shift relative to the planned FUS trajectory) and the angle relative to the skull. The lateral and axial shift ranged between 0.9 mm to 6.4 mm and 1.0 mm to 7.6 mm. When targeting the caudate, the overall shift ranged between 2.4 mm to 6.5 mm with an average of 3.7 mm, and the angle shift was 8.1° with an averaged incidence angle of 5.0° and refraction angle of 7.1°. When targeting the putamen, it ranged between 4.4 mm to 10.1 mm with an average of 7.5 mm, and the angle shift was 7.3° with an averaged incidence angle of 14.2° and refraction angle of 15.8°.

The effect of the incidence angle on the cavitation monitoring (Fig. 6D) was investigated by performing PCD calibration in NHP 2 with varied incidence angle targeting at basal ganglia (right caudate and right putamen with the same tissue composition in both sonicated region and BBB opening region). The SCD_h_ was used since it was reliably detected at the pressures of 100 kPa to 600 kPa. With a lower incidence angle targeting at the right caudate (incidence angle: 6.6°), the SCD_h_ was higher than that for the right putamen (incidence angle: 21.8°). However, the difference was statistically insignificant due to the intra-animal variation, suggesting an insignificant effect of transducer orientation to cavitation monitoring.

### Safety

**Figure 7:**
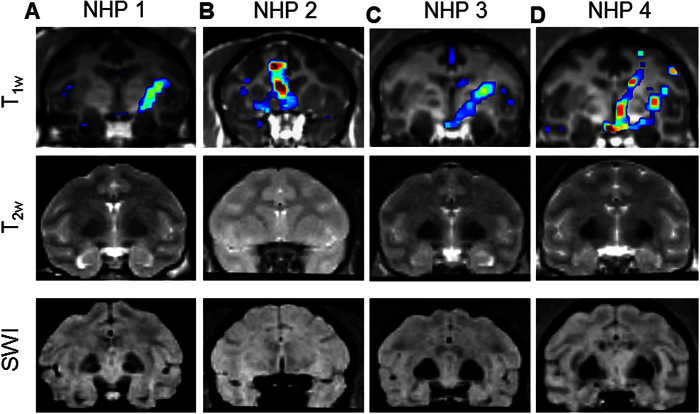
Safety assessments in the MRI. (**A**) NHP 1 targeting putamen at 275 kPa. (**B**) NHP 2 targeting caudate nucleus and putamen at 400 kPa. (**C**) NHP 3 targeting putamen at 450 kPa. (**D**) NHP 4 targeting caudate nucleus and putamen at 600 kPa. No edema or hemorrhage was detected in any of the cases. 1^st^ row: contrast enhancement of the BBB opening overlaying onto the post-Gd T_1w_ imaging; 2^nd^ row: T_2w_ imaging for edema; 3^rd^ row: susceptibility-weighted imaging (SWI) for hemorrhage.

The safety of the FUS procedure was evaluated in all experiments using MRI in 1 h (edema in hyper-intensity of T_2w_ imaging, and hemorrhage in hypo-intensity of SWI). No edema, hemorrhage, or any kind of macroscopic damage was detected in any of the animal in this study. Representative cases with large BBB openings in the four animals were shown in Fig. 7. For NHP 1, one sonication was performed at 275 kPa (putamen, opening volume: 397 mm^3^), two for NHP 2 at 400 kPa (caudate and putamen, opening volume: 783 mm^3^ and 436 mm^3^, respectively), one for NHP 3 at 450 kPa (putamen, opening volume: 845 mm^3^), and two for NHP 4 at 600 kPa (caudate and putamen, opening volume: 623 mm^3^ and 539 mm^3^, respectively). In addition, no damage was detected in the studies of the vasculature effect (Fig. 5) despite the high cavitation response. The damage was not detectable but, if present, might be microscopic and insignificant in histological examination for sonications without detectable damage in MRI^14^.

## Discussion

Our findings presented herein have provided a link between the transcranial cavitation detection for assessment of the BBB opening and the drug delivery outcomes in non-human primates through the use of both stable and inertial cavitation dose. The findings demonstrated a clear relationship between the cavitation measures and the BBB opening characteristics in non-human primates such as the opening volume, the drug amount delivered and the delivery efficiency. Cavitation was detected in both gray and white matter and was correlated with the BBB opening in both, while the cavitation response varied by the large cerebral vessels due to the change of cavitation threshold.

Several new findings have been shown in this study in comparison to previous NHP studies. First, cavitation monitoring was shown to be highly correlated with clinically relevant measures in the deeply seated subcortical structures, an improvement compared with the previous study using relative MRI enhancement in the cerebral cortex^32^. Second, cavitation monitoring was capable of detecting the delivery in both gray and white matter, while large vessels (diameter >1 mm) resulted in saturated stable cavitation response and significantly higher inertial cavitation response at high pressures without detectable damage in the MRI. Third, the cavitation quantification using harmonics, ultraharmonics, and broadband emission could improve the correlation to the BBB opening and drug delivery via compensation of the effect of brain heterogeneity and the nonlinear skull effect. Although harmonics can be more easily detected, they could be hindered by nonlinear skull or large vessel effects at higher pressures and deteriorate the correlation with drug delivery and BBB opening. The primate skull is reported to contribute to harmonic signals at higher pressures, which decreased the cavitation signal-to-noise ratio^31^. Furthermore, harmonics from bubbles in the large vessels could reach a plateau at higher pressures (Fig. 5). These could be the reason why the use of harmonics has previously failed in predicting the BBB opening volume in NHP^33^, and that harmonics were correlated well mostly in the cerebral cortex in a small juvenile monkey with fewer large vessels and thinner skull producing insignificant skull effects^32^.

Inertial cavitation reported to be indicative of vascular damage^15^ or using ultraharmonics to control the treatment^28^ should be considered with caution in large animals, since the large vessels can result in high ultraharmonics and broadband emission without detected damage in radiologic examination implying no significant damage in the histological examination^15^. This vascular effects in humans could prove even more important due to the larger sizes of cerebral vessels compared to smaller animals (e.g. the cerebral artery diameter for mice, monkey, and human is 0.2 mm^35^, 1.2 mm^36^, and 2.0 mm^37^, respectively). The reason behind variable cavitation monitoring may lie in the fact that the microbubbles circulating in large vessels responded differently to FUS. As has been reported previously, the threshold of inertial cavitation was lower in larger vessels^38^,^39^, so more bubbles may be disrupted in large vessels without stable cavitation, resulting in the decrease of SCD_h_ and SCD_u_ at higher pressures with a monotonically increase of the ICD. Since larger vessels allow microbubbles to nonlinearly oscillate, the energy of volumetric oscillation (SCD_h_) may convert to shell oscillation (SCD_u_) and violent bubble oscillation leading to bubble collapse (ICD)^21^. The high broadband emission in this case may not cause vascular damage as the microjets may exert negligible forces towards the vessel wall due to the larger lumen space. Furthermore, large vessels composed of thicker walls and muscles may not easily be disrupted. Therefore, large vessels should be excluded from the FUS focal region for reliable cavitation monitoring.

For molecular delivery to the heterogeneous brain, the BBB opening results showed a localized delivery in both the contrast enhancement and the [*Gd*]_*c*_ map (Fig. 2). The highest [*Gd*]_*c*_ after BBB opening was the same as that of the epicranial muscle (Table 1), since the highest tissue permeability after BBB opening was comparable to that of tissue without BBB^40^. Moreover, the highest delivery ([*Gd*]_*c*_) occurred only at the center of the focus given a larger opening volume (298 or 393 mm^3^) with a shape similar to that of the transducer focus. This suggests that the technique is suitable for region- or point-specific delivery with the highest efficiency, and the localization can be further improved using chirp coded acoustic pulses^41^. If a higher amount of delivery is required, increasing the injection dosage of the drugs could enhance the amount of delivery^42^. If a larger opening volume is desired, sonication at multiple locations would be necessary. However, the BBB opening occurred with greater ease in the gray than in the white matter^15^ was confirmed in this study. BBB opening occurred in both gray and white matter with the probability in the gray matter three times higher than that for the white matter (Fig. 4), and the drug concentration (Gd) was significantly higher in the gray matter as well (Fig. 2). This may be due to the lower vascularity or higher attenuation in the white matter than that in the gray matter^43^. Moreover, since cavitation could be monitored in both tissue types, this discrepancy may be associated with blood perfusion differences during sonication. For example, the blood volume (1.5–1.8 times^44^,^45^) or blood flow (1.7 times^46^) in the gray matter was higher than that of the white matter. Future studies regarding this issue would be required in optimizing this technique for various tissue types.

In addition, the BBB opening was achieved targeting a region including the large vessel MCA (Fig. 5). However, since microbubbles in circulation would attenuate ultrasound wave propagation at varying extents depending on the vessel distribution, size, and microbubble concentration^47^, this microbubble shielding effect may impose potential difficulties in humans^17^ as it was reported to hinder BBB opening in monkeys^14^. Therefore, large cerebral blood vessels should be avoided in the targeted region or acoustic path during FUS planning in order to avoid opening failure as well as monitoring discrepancies. MRA could be a useful tool for visualizing the position and size of cerebral blood vessels during the pre-planning process. Furthermore, the use of nanodroplets to induce BBB opening could potentially minimize the shielding effect since nanodroplets vaporize into bubbles only in the focus and induce opening, which also strongly decreases variability in the relationship of cavitation dose to BBB opening^27^.

Modeling bubble dynamics could potentially provide a priori knowledge for the safety and efficacy of BBB opening and drug delivery in various tissue types, as the mechanical effects of cavitation have long thought to associate with vascular permeability enhancement. A proper model is thus required for these emerging therapeutic purposes. Currently, modified or lumped-parameter models based on the Rayleigh-Plesset equation (free gas bubble model) have enabled us to investigate the behavior of coated microbubbles^48^,^49^ and the bubble-microvessel wall effects^50^, assuming symmetric bubble oscillating in free space. However, a finite-element modelling (FEM) approach provides flexibility for both symmetric and asymmetric bubble oscillation while considering a variety of bubble and *in vivo* environmental properties^51^. Despite the findings obtained with this modelling approach, many efforts are also required to build a linkage to the *in vivo* BBB opening and drug delivery. First, the mechanical effects directly associated with the therapeutic effects need to be identified. Second, the *in vivo* microbubble properties must be measured, including the size, shell^25^,^52^, stiffness^53^, and distribution of bubbles as well as the shape, size, and wall elasticity of the microvessels. Third, the bubble-to-bubble interaction and the bubble behavior after rupture^49^.

In this study, the targeting shift in the putamen was higher than in the caudate (Fig. 6). Both the gyrencephalic brain and the incidence angle could play an important role as more layers of gyrus and sulcus were included in the beam path when targeting the putamen with larger incidence angles. Heterogeneous tissue in the acoustic beam path may affect the targeting accuracy due to the refraction between layers and the heterogeneous BBB opening in the gray and white matter affecting the analysis. Higher incidence angles could cause larger shifts due to the wave distortion in the skull^54^ and refraction between the skull layer as well (refraction angle > incidence angle). Besides, the stereotactic arm holding the heavy FUS transducer may cause shift while targeting with a large angle deviating from the mid-sagittal plane.

## Conclusion

The study presented herein expanded the role of transcranial cavitation monitoring in NHPs *in vivo* and demonstrated a correlation with the BBB opening volume and the drug delivery efficiency with various the tissue types and targeting. The achieved BBB opening volume, amount of delivered molecules and delivery efficiency was as high as 800 mm^3^, 40 nmol, and 0.004%, respectively, and could be predicted by the real-time cavitation monitoring. Quantitative cavitation monitoring and drug delivery were achieved in both the gray and white matter, with the probability of successful BBB opening three times higher in the gray matter than in the white matter. The average targeting shift was 3.7 mm in the caudate and 7.5 mm in the putamen, and the incidence angle to the skull had negligible effects on the cavitation monitoring, showing the capability of cavitation monitoring. The large cerebral vessels, however, may affect cavitation monitoring, and should be avoided in different targets.

## Methods

### Preparation of Animals

In accordance with the National Institutes of Health Guidelines for animal research, all procedures were reviewed and approved by the Institutional Animal Care and Use Committee at Columbia University and the New York State Psychiatric Institute. Four male rhesus macaques (Macaca mulatta, weight: 7–10 kg, age: 8–20 yo) were used in this study. Each animal was sedated with ketamine (5–15 mg/kg in conjunction with 0.04 mg/kg of atropine through intramuscular injection) for placement of an endotracheal tube and an intravenous catheter in the saphenous vein, and was under anesthesia using 1–2% isoflurane-oxygen mixture with vital signs (electrocardiography, heart rate, blood pressure, SpO_2_, breathing rate, end-tidal CO_2_) monitored during the entire experiments. No animals were euthanized in this study.

### Ultrasound System

The system setup is shown in Supplementary Fig. S1. For sonication with FUS, a spherically-focused single-element transducer (H-107, Sonic Concepts, WA, USA) operating at 0.5 MHz (full-width-at-half-maximum focal size: 5.85 mm in width and 34 mm in depth, geometric focal depth: 62.6 mm) was used. For real-time monitoring of the acoustic cavitation emission (passive cavitation detection, PCD), a spherically focused, flat-band, polyvinylidene fluoride (PVDF) hydrophone (Y-107, Sonic Concepts, WA, USA), coaxially and confocally aligned with the FUS transducer, served as the passive cavitation detector. A PC workstation (model T7600, Dell) with a customized program in MATLAB^®^ (Mathworks, MA, USA) was developed to automatically control the sonication through a programmable function generator (model 33220 A, Agilent Technologies, CA, USA) and a 50-dB amplifier (A075, ENI, NY, USA). The PCD signal acquisition was performed with a 14-bit analog-to-digital converter (Gage Applied Technologies, QC, Canada) (sampling rate: 50 MHz) after 20-dB amplification. The PCD signals were monitored transcranially in real time including the frequency spectra and cavitation doses, which were reported to be detectable with the same system and method at pressure as low as 100 kPa *in vivo* through the NHP skull^31^.

### Experimental Procedures

A stereotaxis-based method developed previously^33^ was used for targeting the dorsal striatum (caudate and putamen) (Supplementary Fig. S1), deep subcortical structures associated with neurodegenerative diseases such as Parkinson’s disease. The pressures at the focus of the FUS transducer were calibrated using a bullet hydrophone through *ex vivo* rhesus macaque skulls, and 50% of pressure loss due to the skull was compensated in the *in vivo* experiments^55^. The in-house made, lipid-shelled, monodisperse microbubbles (4–5 μm in diameter)^56^ were freshly diluted to 3 mL with a dosage of 2.5 × 10^8^ bubbles/kg of body weight. Another 3 mL of saline was used to flush after microbubble injection. At the beginning of the FUS procedure, the control sonication (duration = 5 s) was performed before microbubble injection as a baseline for cavitation monitoring, and then the microbubbles were injected in a bolus intravenously (saphenous vein) followed by saline flush within 30 s while the sonication started at the same time (peak negative pressure = 200–600 kPa, pulse length = 10 ms, pulse-repetition frequency = 2 Hz, duration = 2 min). A second sonication was performed in 11 out of a total of 47 experiments at a second non-overlapping target 20 min after the microbubbles were eliminated from the first experiment.

In order to investigate the effects of targeting and heterogeneous brain, PCD calibration (N = 31) (a 5-s consecutive sonication at monotonically increasing pressures ranging within 100–600 kPa starting at 10 s after re-injecting half the dose of microbubbles)^31^ was performed after the regular sonication targeting a region proximal to the medial cerebral artery (MCA). After the FUS procedure, the animal was transferred to the MRI suite for assessing the BBB opening and safety within 1 h. Two sham cohorts were also performed by applying FUS without injecting microbubbles (FUS +/MB−, N = 4) or without FUS procedure (FUS−/MB−, N = 3) for comparison to determine BBB opening in the experimental groups.

### Magnetic Resonance Imaging Acquisition

A 3.0 T MRI system (Achieva, Philips Medical Systems, USA) with an eight-channel head coil was used for assessing safety and BBB opening. T_1w_ imaging was used for BBB opening detection and opening volume quantification because of its higher sensitivity, while T_1_ mapping was for quantifying the amount of delivered MR contrast agents and the delivery efficiency. For assessing BBB opening, both pre- and post- contrast agent T_1_-weighted (T_1w_) images and T_1_ maps using the variable flip-angle (VFA) SPGR method were acquired. The contrast agent used in this study was Gd-DTPA-BMA as the model drug (gadodiamide or Gd, molecular weight = 573.66 Da; Omniscan^®^, GE Healthcare, NJ, USA) with the same dosage suggested for patients (0.2 mL/kg or 0.1 mmol/kg of body weight). Pre- and post-Gd (40 min after injection) T_1w_ images using 3D spoiled gradient echo (SPGR) sequence (TR/TE = 8.5/4.8 ms, FA = 8°, SR = 0.97 × 0.97 × 1 mm^3^) were acquired for detecting the opening and analyzing the opening volume. For quantitative analysis of the Gd concentration and delivery efficiency, pre- and post-Gd (20 min after injection) T_1_ maps were acquired using a series of 3D SPGR sequence with five flip angles (TR/TE = 10/4 ms, FAs = 5°/10°/15°/20°/35°, SR = 0.89 × 0.89 mm^2^, SL = 1 mm). For detecting edema, T_2_-weighted (T_2w_) images were acquired using 3D Turbo Spin Echo sequence (TR/TE = 3000/80 ms, flip angle or FA = 90°, resolution = 0.42 × 0.42 × 2 mm^3^). For detecting hemorrhage, susceptibility-weighted imaging (SWI) was performed (TR/TE = 19/27 ms, FA = 15°, resolution = 0.44 × 0.44 × 1 mm^3^). Magnetic resonance angiography (MRA) was conducted in order to visualize the size and orientation of blood vessels using 3D time-of-flight SPGR sequence (TR/TE = 23/3.4 ms, FA = 15°, resolution = 0.89 × 0.89 × 1 mm^3^) in a separate 3.0 T scanner (Signa, GE Healthcare, USA) with a customized two-channel head coil.

### Quantification for BBB Opening

The BBB opening volume was quantified using pre- and post-Gd T_1w_ images in Matlab with custom-built programs^31^. In brief, both pre- and post-Gd images were first registered to the individual stereotactically-aligned images (IST) with FSL’s FLIRT toolbox^57^, computing the ratio of post- to pre-Gd images as a measurement of contrast enhancement, which was normalized by linear scaling with reference to the unsonicated thalamus and the anterior cerebral artery (ACA) as shown in dashed and solid circle in the horizontal slice (Fig. 1A), respectively. In order to filter out the background contrast enhancement in the cerebral vessels and muscle tissue outside the brain for quantifying the BBB opening volume, the brain mask was applied (generated using pre-Gd T_1w_ images from the no-FUS sham cohort with FSL’s Brain Extraction Toolbox^58^) and the enhancement images of the FUS−/MB- sham cohort for each individual was subtracted from the enhancement images (coronal slice in Fig. 1A), giving rise to Fig. 1B. Finally, the opening volume was calculated by applying a volume of interest (VOI, 10 × 10 × 32.5 mm^3^) on the targeted region (solid box) subtracting the VOI on the contralateral side (dashed box) as shown in Fig. 1C. The threshold of BBB opening (80 mm^3^) was defined by the average opening volume plus 3 times the standard deviation in the FUS + /MB− sham cohort.

Similarly, the Gd concentration maps^42^,^59^ provide quantification of the Gd amount that crossed the BBB. The delivery efficiency was calculated using the pre- (Fig. 1D) and post-Gd T_1_ maps (Fig. 1E)^59^. First, the standard line fit method of VFA SPGR^60^ was used to calculate the pre- and post-T_1_ maps after registering the 3D SPGR images of various flip angles to the IST. Then, the Gd concentration map (Fig. 1F) was generated based on the following equation:


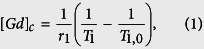


where [*Gd*]_*c*_ is the Gd concentration, *r*_1_ is the relaxivity of the contrast agents (4 s^−1^mM^−1^ for Gd-DTPA-BMA^61^), *T*_1_ is the post-Gd T_1_ time, and *T*_1,0_ is the pre-Gd T_1_ time. Note that in the [*Gd*]_*c*_ map (Fig. 1F), the concentrations in the unopened brain tissue (thalamus, dashed circle), temporalis muscle (black solid circle), and anterior cerebral vessels (ACA, white solid circle) were also calculated and compared with that of the BBB opening area. In order to filter out the [*Gd*]_*c*_ in the cerebral vessels and muscle tissue outside the brain for quantifying the delivered Gd due to BBB opening, the [*Gd*]_*c*_ maps (coronal slice in Fig. 1F) were thresholded by setting the concentration higher than that in ACA to zero and applying the brain mask, giving rise to Fig. 1G. Finally, the amount of delivered Gd was calculated based on equation (2) by applying a volume of interest (VOI, 10 × 10 × 32.5 mm^3^) on the targeted region (*VOI*_*ipsi*_, solid box in Fig. 1H) subtracting the VOI on the contralateral side (*VOI*_*contra*_, dashed box in Fig. 1H) for excluding the intrinsic Gd retention:


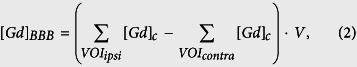


where [*Gd*]_*BBB*_ is the amount of delivered Gd (mole) and *V* is the voxel volume in the T_1_ map. The delivery efficiency was defined as the percentage of the amount of delivered Gd to the total amount of injected Gd (estimated to be 1 mmol).

The opening volume was separated by the tissue type (i.e., the gray matter, white matter, blood, cerebral spinal fluid) segmented based on the T_1_ time in the pre-Gd T_1_ map. The T_1_ time for blood is between 1 ms to 700 ms, 700 ms to 1170 ms for white matter, 1170 ms to 1800 ms for gray matter, and 1800 ms to 5000 ms for cerebral spinal fluid based on our measurement in the NHP and the previous study in humans^60^.

### Analysis of targeting accuracy

The targeting accuracy was analyzed by comparing the planned targeting to the quantified BBB opening results using the T_1w_ images as described in the previous section. The planned targeting was composed of a targeting vector with a focus and an approaching direction (trajectory) for an interested brain structure, as well as the angle of incidence to the skull. This incidence angle was defined as the angle between the targeting vector and the vector normal to the outer surface of the skull (Supplementary Fig. 2), which was estimated using the 3-D gradient field in the skull mask after the skull extraction on the pre-Gd T_1w_ images with the Brain Extraction Toolbox in FSL^62^. The BBB opening vector was subsequently defined by the 3-D center of mass in the BBB opening in the VOI described previously and the vector from the 3D linear curve fitting of the 2-D center of mass in each of the horizontal slices, respectively. Similar to the incidence angle, the refraction angle was the angle between the BBB opening vector and the vector normal to the inner layer of the skull in the 3D gradient field. Finally, the target shift was defined as the distance between the center of focus in the targeting vector and the center of mass in the BBB opening, and the lateral and axial shift was the shift perpendicular and parallel to the targeting vector, respectively.

### Quantification of Acoustic Cavitation Emission

Based on the method previously proposed^31^, stable cavitation dose with harmonics (SCD_h_), stable cavitation dose with ultraharmonics (SCD_u_), and the inertial cavitation dose (ICD) representing volumetric oscillation, shell oscillation, and drastic bubble oscillation to bubble collapse, respectively, were quantified separately. The frequency spectrum of the PCD signal (in volts) from each pulse (1.25–5.00 MHz) was separately filtered to extract the harmonics (maxima in the bandwidth of 20 kHz around the harmonic frequency n*f, where f = 0.5 MHz and n = 3, 4, 5, …. 10), ultraharmonics (maxima in the bandwidth of 20 kHz around the ultraharmonic frequency m/2*f, where f = 0.5 MHz and m = 5, 7, 9, …. 19), and broadband emissions (signal after suppressing the harmonics and the ultraharmonics; 360 kHz around the harmonic frequency and 100 kHz around the ultraharmonic frequency, respectively), and the root-mean-squared (RMS) amplitude of the filtered PCD signal from one pulse (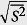) was defined as the cavitation level (*dCD*, representing *dSCD*_*h*_, *dSCD*_*u*_ or *dICD*), i.e., the cavitation dose of a single pulse. The cavitation dose of the entire sonication (*CD*, representing either *SCD*_*h*_, *SCD*_*u*_ or *ICD*) was thus equal to the sum of the cavitation levels over the entire sonication time:


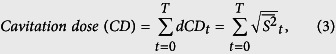


where *t* is the time for each pulse and *T* the total sonication duration.

### Statistical Analysis

The two-tailed Student’s t-test or an unsupervised linear regression fit were performed using GraphPad Prism (La Jolla, CA, USA) for quantitative PCD in assessing the BBB opening outcomes. Statistical significance was defined with p-value < 0.05.

## Additional Information

**How to cite this article**: Wu, S.-Y. *et al.* Characterizing Focused-Ultrasound Mediated Drug Delivery to the Heterogeneous Primate Brain *In Vivo* with Acoustic Monitoring. *Sci. Rep.* **6**, 37094; doi: 10.1038/srep37094 (2016).

**Publisher’s note:** Springer Nature remains neutral with regard to jurisdictional claims in published maps and institutional affiliations.

## Supplementary Material

Supplementary Information

Supplementary Dataset

## Figures and Tables

**Table 1:** Gd retention in different tissues after Gd injection (without applying FUS).

	Thalamus (mM)	Muscle (mM)	ACA* (mM)
NHP 2	0.017 ± 0.005	0.071 ± 0.008	0.153 ± 0.022
NHP 3	0.012 ± 0.004	0.060 ± 0.009	0.142 ± 0.014

^*^Anterior cerebral artery.

## References

[b1] Pardridge W. M. Drug transport across the blood-brain barrier. J Cereb Blood Flow Metab 32, 1959–1972 (2012). 22929442 10.1038/jcbfm.2012.126 PMC3494002

[b2] Abbott N. J., Ronnback L. & Hansson E. Astrocyte-endothelial interactions at the blood-brain barrier. Nature Reviews Neuroscience 7, 41–53 (2006). 16371949 10.1038/nrn1824

[b3] Timbie K. F., Mead B. P. & Price R. J. Drug and gene delivery across the blood-brain barrier with focused ultrasound. J Control Release (2015). 10.1016/j.jconrel.2015.08.059 PMC4656107 26362698

[b4] Liu H. L. *et al.* Blood-brain barrier disruption with focused ultrasound enhances delivery of chemotherapeutic drugs for glioblastoma treatment. Radiology 255, 415–425 (2010). 20413754 10.1148/radiol.10090699

[b5] Kovacs Z. *et al.* Prolonged survival upon ultrasound-enhanced doxorubicin delivery in two syngenic glioblastoma mouse models. J Control Release 187, 74–82 (2014). 24878186 10.1016/j.jconrel.2014.05.033

[b6] Burgess A. *et al.* Alzheimer disease in a mouse model: MR imaging-guided focused ultrasound targeted to the hippocampus opens the blood-brain barrier and improves pathologic abnormalities and behavior. Radiology 273, 736–745 (2014). 25222068 10.1148/radiol.14140245 PMC4314115

[b7] Leinenga G. & Gotz J. Scanning ultrasound removes amyloid-beta and restores memory in an Alzheimer’s disease mouse model. Sci Transl Med 7, 278ra233 (2015). 10.1126/scitranslmed.aaa2512 25761889

[b8] Wang S., Olumolade O. O., Sun T., Samiotaki G. & Konofagou E. E. Noninvasive, neuron-specific gene therapy can be facilitated by focused ultrasound and recombinant adeno-associated virus. Gene therapy 22, 104–110 (2015). 25354683 10.1038/gt.2014.91 PMC4294560

[b9] Thévenot E., Jordão J. F., O’Reilly M. A., Markham K., Weng Y.-Q., Foust K. D., Kaspar B. K., Hynynen K. & Aubert I. Targeted Delivery of Self-Complementary Adeno-Associated Virus Serotype 9 to the Brain, Using Magnetic Resonance Imaging-Guided Focused Ultrasound. Human Gene Therapy 23(11), 1144–1155 (2012). 22838844 10.1089/hum.2012.013 PMC3498907

[b10] Nance E. *et al.* Non-invasive delivery of stealth, brain-penetrating nanoparticles across the blood-brain barrier using MRI-guided focused ultrasound. J Control Release 189, 123–132 (2014). 24979210 10.1016/j.jconrel.2014.06.031 PMC4125545

[b11] Mead B. P. *et al.* Targeted gene transfer to the brain via the delivery of brain-penetrating DNA nanoparticles with focused ultrasound. J Control Release 223, 109–117 (2016). 26732553 10.1016/j.jconrel.2015.12.034 PMC4739627

[b12] Burgess A. *et al.* Targeted delivery of neural stem cells to the brain using MRI-guided focused ultrasound to disrupt the blood-brain barrier. PLoS ONE 6, e27877 (2011). 22114718 10.1371/journal.pone.0027877 PMC3218061

[b13] Marquet F., Tung Y. S., Teichert T., Ferrera V. P. & Konofagou E. E. Noninvasive, transient and selective blood-brain barrier opening in non-human primates *in vivo*. PLoS ONE 6, e22598 (2011). 21799913 10.1371/journal.pone.0022598 PMC3142168

[b14] Tung Y. S., Marquet F., Teichert T., Ferrera V. & Konofagou E. E. Feasibility of noninvasive cavitation-guided blood-brain barrier opening using focused ultrasound and microbubbles in nonhuman primates. Appl Phys Lett 98, 163704 (2011). 21580802 10.1063/1.3580763 PMC3094460

[b15] McDannold N., Arvanitis C. D., Vykhodtseva N. & Livingstone M. S. Temporary disruption of the blood-brain barrier by use of ultrasound and microbubbles: safety and efficacy evaluation in rhesus macaques. Cancer research 72, 3652–3663 (2012). 22552291 10.1158/0008-5472.CAN-12-0128 PMC3533365

[b16] Downs M. E. *et al.* Long-Term Safety of Repeated Blood-Brain Barrier Opening via Focused Ultrasound with Microbubbles in Non-Human Primates Performing a Cognitive Task. PLoS ONE 10, e0125911 (2015). 25945493 10.1371/journal.pone.0125911 PMC4422704

[b17] Carpentier A. *et al.* Clinical trial of blood-brain barrier disruption by pulsed ultrasound. Sci Transl Med 8, 343re342 (2016). 10.1126/scitranslmed.aaf6086 27306666

[b18] Leinenga G., Langton C., Nisbet R. & Gotz J. Ultrasound treatment of neurological diseases-current and emerging applications. Nat Rev Neurol 12, 161–174 (2016). 26891768 10.1038/nrneurol.2016.13

[b19] McDannold N., Vykhodtseva N. & Hynynen K. Targeted disruption of the blood-brain barrier with focused ultrasound: association with cavitation activity. Phys Med Biol 51, 793–807 (2006). 16467579 10.1088/0031-9155/51/4/003

[b20] Leighton T. G. The Acoustic Bubble (1994).

[b21] Stride E. Physical principles of microbubbles for ultrasound imaging and therapy. Cerebrovascular diseases (Basel, Switzerland) 27 Suppl 2, 1–13 (2009). 10.1159/000203122 19372656

[b22] Krasovitski B. & Kimmel E. Shear stress induced by a gas bubble pulsating in an ultrasonic field near a wall. IEEE transactions on ultrasonics, ferroelectrics, and frequency control 51, 973–979 (2004). 10.1109/tuffc.2004.1324401 15344403

[b23] Chen H., Kreider W., Brayman A. A., Bailey M. R. & Matula T. J. Blood vessel deformations on microsecond time scales by ultrasonic cavitation. Phys Rev Lett 106, 034301 (2011). 21405276 10.1103/PhysRevLett.106.034301 PMC3087441

[b24] Sheikov N., McDannold N., Sharma S. & Hynynen K. Effect of focused ultrasound applied with an ultrasound contrast agent on the tight junctional integrity of the brain microvascular endothelium. Ultrasound in medicine & biology 34, 1093–1104 (2008). 18378064 10.1016/j.ultrasmedbio.2007.12.015 PMC2518085

[b25] Tung Y. S., Vlachos F., Feshitan J. A., Borden M. A. & Konofagou E. E. The mechanism of interaction between focused ultrasound and microbubbles in blood-brain barrier opening in mice. J Acoust Soc Am 130, 3059–3067 (2011). 22087933 10.1121/1.3646905 PMC3248062

[b26] Wu S. Y., Chen C. C., Tung Y. S., Olumolade O. O. & Konofagou E. E. Effects of the microbubble shell physicochemical properties on ultrasound-mediated drug delivery to the brain. J Control Release 212, 30–40 (2015). 26065734 10.1016/j.jconrel.2015.06.007 PMC4527345

[b27] Chen C. C. *et al.* Targeted drug delivery with focused ultrasound-induced blood-brain barrier opening using acoustically-activated nanodroplets. J Control Release 172, 795–804 (2013). 24096019 10.1016/j.jconrel.2013.09.025 PMC3866692

[b28] O’Reilly M. A. & Hynynen K. Blood-Brain Barrier: Real-time Feedback-controlled Focused Ultrasound Disruption by Using an Acoustic Emissions-based Controller. Radiology 263, 96–106 (2012). 22332065 10.1148/radiol.11111417 PMC3309801

[b29] Tsai C. H., Zhang J. W., Liao Y. Y. & Liu H. L. Real-time monitoring of focused ultrasound blood-brain barrier opening via subharmonic acoustic emission detection: implementation of confocal dual-frequency piezoelectric transducers. Phys Med Biol 61, 2926–2946 (2016). 26988240 10.1088/0031-9155/61/7/2926

[b30] Sun T. *et al.* Acoustic cavitation-based monitoring of the reversibility and permeability of ultrasound-induced blood-brain barrier opening. Phys Med Biol 60, 9079–9094 (2015). 26562661 10.1088/0031-9155/60/23/9079 PMC4668271

[b31] Wu S. Y. *et al.* Transcranial cavitation detection in primates during blood-brain barrier opening–a performance assessment study. IEEE Trans Ultrason Ferroelectr Freq Control 61, 966–978 (2014). 24859660 10.1109/TUFFC.2014.2992 PMC4034133

[b32] Arvanitis C. D., Livingstone M. S., Vykhodtseva N. & McDannold N. Controlled ultrasound-induced blood-brain barrier disruption using passive acoustic emissions monitoring. PLoS ONE 7, e45783 (2012). 23029240 10.1371/journal.pone.0045783 PMC3454363

[b33] Marquet F. *et al.* Real-time transcranial monitoring of safe blood-brain barrier opening in non-human primates. PLoS ONE 9, e84310 (2014). 24505248 10.1371/journal.pone.0084310 PMC3914779

[b34] Wu S.-Y., Aurup C., Sierra Sanchez C., Karakatsani M. E., Grondin J., Zheng W., Ferrera V. P. & Konofagou E. E. Computer-aided transcranial ultrasound for time-efficient blood-brain barrier opening, In preparation.

[b35] Schambach S. J. *et al.* Ultrafast high-resolution *in vivo* volume-CTA of mice cerebral vessels. Stroke 40, 1444–1450 (2009). 19213951 10.1161/STROKEAHA.108.521740

[b36] Kapoor K., Kak V. K. & Singh B. Morphology and comparative anatomy of circulus arteriosus cerebri in mammals. Anat Histol Embryol 32, 347–355 (2003). 14651482 10.1111/j.1439-0264.2003.00492.x

[b37] Ashwini Chamanahalli Appaji, Shubha R. & Jayanthi Kadaba Srinivasan. Comparative anatomy of the circle of Willis in man, cow, sheep, goat, and pig. Neuroanatomy 7, 54–85 (2008).

[b38] Sassaroli E. & Hynynen K. On the impact of vessel size on the threshold of bubble collapse. Appl Phys Lett 89, 123901 (2006).

[b39] Sassaroli E. & Hynynen K. Cavitation threshold of microbubbles in gel tunnels by focused ultrasound. Ultrasound Med Biol 33, 1651–1660 (2007). 17590501 10.1016/j.ultrasmedbio.2007.04.018 PMC2078601

[b40] Vlachos F., Tung Y. S. & Konofagou E. E. Permeability assessment of the focused ultrasound-induced blood-brain barrier opening using dynamic contrast-enhanced MRI. Phys Med Biol 55, 5451–5466 (2010). 20736501 10.1088/0031-9155/55/18/012 PMC4005850

[b41] Kamimura H. A. S. *et al.* Chirp-and random-based coded ultrasonic excitation for localized blood-brain barrier opening. Phys Med Biol. 60, 7695–712 (2015). 26394091 10.1088/0031-9155/60/19/7695 PMC4580262

[b42] Marty B. *et al.* Dynamic study of blood-brain barrier closure after its disruption using ultrasound: a quantitative analysis. J Cereb Blood Flow Metab 32, 1948–1958 (2012). 22805875 10.1038/jcbfm.2012.100 PMC3463875

[b43] Duck F. A. In Physical properties of tissue: a comprehensive reference book, Edn. illustrated 73–135 (Academic Press, 1990).

[b44] Bulte D., Chiarelli P., Wise R. & Jezzard P. Measurement of cerebral blood volume in humans using hyperoxic MRI contrast. Journal of Magnetic Resonance Imaging 26, 894–899 (2007). 17896390 10.1002/jmri.21096

[b45] Hamberg L. M. *et al.* Measurement of cerebral blood volume with subtraction three-dimensional functional CT. AJNR Am J Neuroradiol 17, 1861–1869 (1996). 8933870 PMC8337543

[b46] Ellenbogen R. G. & Sekhar L. N. Edn. 3 (Elsevier Inc., 2012).

[b47] Tang M. X., Eckersley R. J. & Noble J. A. Pressure-dependent attenuation with microbubbles at low mechanical index. Ultrasound Med Biol 31, 377–384 (2005). 15749561 10.1016/j.ultrasmedbio.2004.12.009

[b48] Doinikov A. A. & Bouakaz A. Review of shell models for contrast agent microbubbles. IEEE Trans Ultrason Ferroelectr Freq Control 58, 981–993 (2011). 21622054 10.1109/TUFFC.2011.1899

[b49] Faez T. *et al.* 20 years of ultrasound contrast agent modeling. IEEE Trans Ultrason Ferroelectr Freq Control 60, 7–20 (2013). 23287909 10.1109/TUFFC.2013.2533

[b50] Qin S. & Ferrara K. W. Acoustic response of compliable microvessels containing ultrasound contrast agents. Phys Med Biol 51, 5065–5088 (2006). 17019026 10.1088/0031-9155/51/20/001 PMC2847449

[b51] Hosseinkhah N. & Hynynen K. A three-dimensional model of an ultrasound contrast agent gas bubble and its mechanical effects on microvessels. Phys Med Biol 57, 785–808 (2012). 22252221 10.1088/0031-9155/57/3/785 PMC3367455

[b52] Chen C. C., Wu S.-Y., Finan J. D., Morrison B. & Konofagou E. An experimental study on the stiffness of size-isolated microbubbles using atomic force microscopy. IEEE Trans Ultrason Ferroelectr Freq Control 60, 524–534 (2013). 23475918 10.1109/TUFFC.2013.2594 PMC4123865

[b53] Sierra C., Acosta C., Chen C., Wu S.-Y., Karakatsani M. E., Bernal M. & Konofagou E. E. Lipid microbubbles as a vehicle for targeted drug delivery using focused ultrasound-induced blood–brain barrier opening. J Cereb Blood Flow Metab June 8 (2016). 10.1177/0271678X16652630 PMC5453447 27278929

[b54] White P. J., Clement G. T. & Hynynen K. Longitudinal and shear mode ultrasound propagation in human skull bone. Ultrasound Med Biol 32, 1085–1096 (2006). 16829322 10.1016/j.ultrasmedbio.2006.03.015 PMC1560344

[b55] Marquet F., Tung Y. S. & Konofagou E. E. Feasibility study of a clinical blood-brain barrier opening ultrasound system. Nano Life 1, 1–14 (2011). 10.1142/S1793984410000286 PMC4031659 24860623

[b56] Feshitan J. A., Chen C. C., Kwan J. J. & Borden M. A. Microbubble size isolation by differential centrifugation. J Colloid Interface Sci 329, 316–324 (2009). 18950786 10.1016/j.jcis.2008.09.066

[b57] Smith S. M. *et al.* Advances in functional and structural MR image analysis and implementation as FSL. Neuroimage 23 Suppl 1, S208–S219 (2004). 15501092 10.1016/j.neuroimage.2004.07.051

[b58] Smith S. M. Fast robust automated brain extraction. Hum Brain Mapp 17, 143–155 (2002). 12391568 10.1002/hbm.10062 PMC6871816

[b59] Samiotaki G., Karakatsani M. E., Wu S.-Y., Buch A. & Konofagou E. Pharmacodynamic analysis and concentration mapping for efficient delivery through the FUS-induced BBB opening in non-human primates *in vivo*. Journal of Therapeutic Ultrasound 3 (2015).

[b60] Liberman Gilad, L. Y. & Bashat Dafna Ben T1 mapping using variable flip angle SPGR data with flip angle correction. Journal of Magnetic Resonance Imaging 40, 171–180 (2014). 24990618 10.1002/jmri.24373

[b61] Sophie Laurent L. V. E. & Robert N. Muller Comparative study of the physicochemical properties of six clinical low molecular weight gadolinium contrast agents. Contrast Media Mol Imaging 1, 128–137 (2006). 17193689 10.1002/cmmi.100

[b62] Jenkinson M., Pechaud M. & Smith S. In Eleventh Annual Meeting of the Organization for Human Brain Mapping (2005).

