# Supplementary material for: Characterizing Focused-Ultrasound Mediated Drug Delivery to the Heterogeneous Primate Brain In Vivo with Acoustic Monitoring
Source: Sci Rep. 2016 Nov 17;6:37094. doi: 10.1038/srep37094 (PMC5112571; doi:10.1038/srep37094)
Supplement: Supplementary Information [file srep37094-s1.pdf]

Characterizing Focused-Ultrasound Mediated Drug Delivery to the Heterogeneous Primate Brain *In Vivo* with Acoustic Monitoring

Shih-Ying Wu[1], Carlos Sierra Sanchez[1], Gesthimani Samiotaki[1], Amanda Buch[1,2], Vincent Ferrera[2], Elisa Konofagou[1,3*]

[1]Department of Biomedical Engineering, Columbia University, New York, New York, USA
[2]Department of Neuroscience, Columbia University, New York, New York, USA
[3]Department of Radiology, Columbia University, New York, New York, USA

*Address correspondence to:
Dr. Elisa Konofagou,
Department of Biomedical Engineering, Columbia University
351 Engineering Terrace, mail code 8904
1210 Amsterdam Avenue, New York, NY, USA
Email: ek2191@columbia.edu
Tel: 212-342-0863
Fax: 212-342-5773

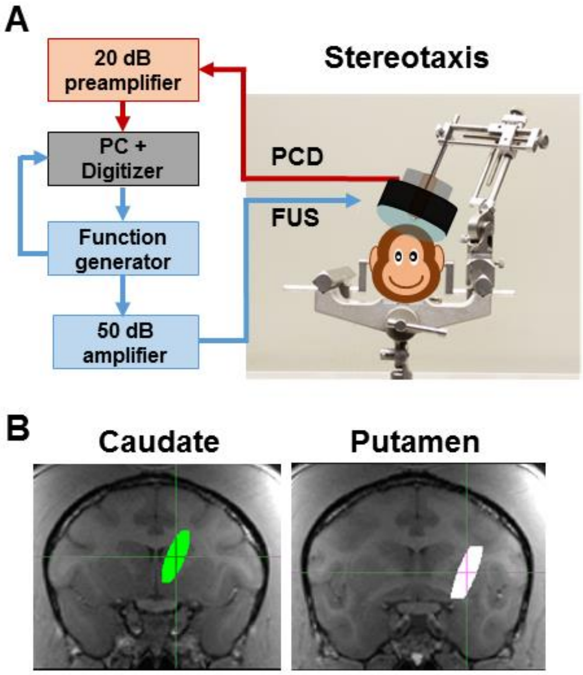

Supplementary Figure 1. Experimental setup (A) and targeted region (B).

(A) A focused ultrasound (FUS) transducer was used for sonication and a hydrophone coaxially and confocally aligned with the FUS transducer served for passive cavitation detection (PCD). (B) Targeting was performed using stereotaxis with pre-planning for focusing at the caudate nucleus (left) or putamen (right) based on the structural MRI scan with a stereotax in advance.

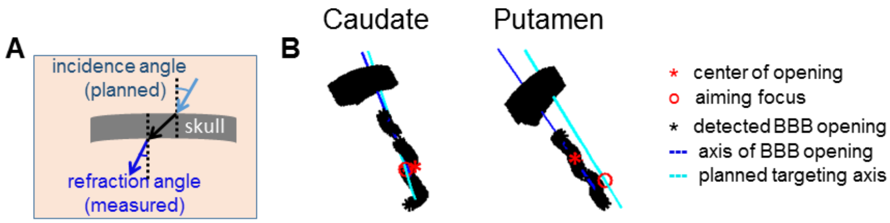

Supplementary Figure 2. Illustration of incidence angle to the skull (A) and the BBB opening in caudate and putamen (B) corresponded to Figure 5A-B. (A) Illustration of acoustic path intersecting the skull, where the incidence angle was measured by the planned targeting trajectory and the refraction angle by the opening trajectory. (B) 3D visualization of targeting and opening trajectory intersecting the skull. Frame-based stereotaxic targeting accuracy.

Supplementary Table 1. Physiological records and vital signs of the animals during sonication

	Age (yo)	Weight (kg)	Heart rate (bpm)	Blood pressure (mmHg)		SpO_2 (%)	Breathing rate (bpm)	$ETCO_2$* (mmHg)
				Systolic	Diastolic			
**NHP 1**	9	9	–	–	–	–	–	–
**NHP 2**	8	8	102 ± 6	70 ± 7	28 ± 4	97 ± 3	15 ± 2	53 ± 2
**NHP 3**	20	10	107 ± 7	61 ± 10	26 ± 2	98 ± 2	11 ± 3	43 ± 3
**NHP 4**	20	10	–	–	–	–	–	–

*end-tidal CO_2
